# Expression of concern on ‘Influence of correct secondary and tertiary RNA folding on the binding of cellular factors to the HCV IRES’

**DOI:** 10.1093/nar/gkad749

**Published:** 2023-09-12

*Nucleic Acids Research*, Volume 28, Issue 4, 15 February 2000, Pages 875–885, https://doi.org/10.1093/nar/28.4.875

The Editors were alerted in August 2023 that two areas in the background of Figure 1B appear more similar than expected.

The Editors have contacted the authors and are investigating. In the interim, we advise readers to examine the details of this study with particular care.

Julian E. Sale, Barry L. Stoddard

Senior Executive Editors

